# Verification of Reference Interval of Thyroid Hormones With Manual and Automated Indirect Approaches: Comparison of Hoffman, KOSMIC and refineR Methods

**DOI:** 10.7759/cureus.39066

**Published:** 2023-05-15

**Authors:** Ashishkumar Agaravatt, Gaurav Kansara, Asha Khubchandani, Hiren Sanghani, Shailesh Patel, Deepak Parchwani

**Affiliations:** 1 Department of Biochemistry, PDU (Pandit Deendayal Upadhyay) Medical College, Rajkot, IND; 2 Department of Biochemistry, Dr. Kiran C. Patel Medical College and Research Institute, Bharuch, IND; 3 Department of Biochemistry, BJ (Byramjee Jeejeebhoy) Medical College, Ahmedabad, IND; 4 Department of Biochemistry, GMERS (Gujarat Medical Education & Research Society) Medical College, Morbi, IND; 5 Department of Biochemistry, Government Medical College, Surat, IND; 6 Department of Biochemistry, All India Institute of Medical Sciences, Rajkot, IND

**Keywords:** thyroid hormone, refiner, kosmic, indirect methods, reference interval

## Abstract

Introduction: The interpretation of quantitative test results requires the availability of appropriate reference intervals (RIs). Every laboratory has been advised by scientific literature and reagent manufacturers to establish RIs for all analytes. Measuring RIs using direct methods is very costly, and it poses ethical and practical challenges. To overcome these challenges, indirect methods, such as Hoffman, and newer automated approaches, such as KOSMIC and refineR, are used to verify RIs for thyroid hormones.

Objective: To verify RIs for thyroid hormones in adult patients using Hoffman, KOSMIC and refineR methods and to compare these with reference ranges given in kit literature or standard textbooks.

Materials and methods: The observed values (results) of thyroid hormone were collected from the LIS (Laboratory Information System) of the Biochemistry Department at the B. J. Medical College and Civil Hospital in Ahmedabad between 1 January 2021 and 31 May 2022. Hoffman, KOSMIC and refineR methods were used to verify the RIs. The computerised Hoffman approach, which Katayev et al. describe, is a simple method for determining RI from hospital data. Zierk et al. pre-validated and suggested the KOSMIC method based on Python programming, whereas refineR was proposed by Tatjana et al. based on R programming language.

Results: Hoffman, KOSMIC and refineR’s indirect RI techniques revealed comparable results with kit literature in free T3 and T4, whereas higher upper reference limits of thyroid-stimulating hormone (TSH) compared to kit literature were observed with KOSMIC and refineR methods. However, the computerised Hoffman method revealed comparable results with TSH also.

Conclusion: Indirect approaches, such as Hoffman, KOSMIC and refineR, provide reliable RI verification for free T3 and T4 utilising patient samples obtained from LIS. However, the manual Hoffman method provides reliable RI verification for TSH data derived from the hospital population as compared to automated approaches, such as KOSMIC and refineR.

## Introduction

Laboratory tests are necessary to evaluate the status of health and guide patient care for people of all age groups. Appropriate reference intervals (RIs) must be available for the interpretation of quantitative test findings [1]. Thyroid hormones (free T3 and T4 and thyroid-stimulating hormone [TSH]) are the most important biomarkers for the diagnosis of various thyroid disorders. It has been demonstrated that RIs for thyroid hormones should be redefined across various countries because of differences in regional iodine consumption and used analytic techniques [2]. RIs also depend on covariates, such as ethnicity, geographic region, sex or age, and appropriate stratification is required. So, all laboratories are advised by scientific literature and reagent manufacturers to establish reference ranges for all analytes by themselves. Measuring RIs using direct methods is very costly with ethical and practical challenges. As an alternative, huge datasets from laboratories made up of both diseased and non-diseased individuals may be utilised to estimate RI using numerous indirect methods [3]. Estimation of reference ranges from retrospective laboratory data is an accepted scientific method. The goal of indirect RIs is to determine the apparent normal distribution findings from a database that contains both diseased and healthy people. Although indirect methods are convenient and inexpensive, it is clear that dealing with a mixed population is more challenging than dealing with a population that is uniformly healthy. As a result, any tool used to distinguish between healthy and diseased distributions must be understood and applied with a high degree of precision [4]. Several indirect techniques have been developed and are now being utilised to estimate RIs, and the most recent and historically significant approaches are the Bhattacharya method [5] and the visual Hoffman approach [6], the truncated minimum likelihood (TML) method developed by Arzideh et al. [7-9], the truncated minimum chi-square method developed by Wosniok et al. [10] and the updated version of the TML technique developed by Zierk et al. called KOSMIC [11] and refineR [12].

The best straightforward method for determining reference range in hospital population is the computerised Hoffman method, as described by Katayev et al. [13], whereas the KOSMIC and refineR newer automated indirect methods have been estimated in numerous benchmarking datasets, demonstrating accurate reference ranges even when samples with a high percentage of pathological test findings were included. Both the newer methods are automated approaches and are freely available as open-source software, which allows future improvement in the method by the scientific community.

The objective of the study is to verify RIs for thyroid hormones in adult patients using Hoffman, KOSMIC and refineR methods and compare them to the reference ranges provided in kit literature or standard textbooks.

## Materials and methods

The observed thyroid hormone values (results) were collected from the LIS (laboratory information system) of the Biochemistry Department at the “B. J. Medical College and Civil Hospital in Ahmedabad” between 1 January 2021 and 31 May 2022. Thyroid hormones were performed on a Beckman Coulter DxI 600 chemiluminescent immunoassay analyzer. Accuracy and precision of results were ensured by following protocols as per ISO 15189:2012-based NABL accreditation. We used three different statistical approaches to estimate the RIs.

The computerised Hoffman approach described by Katayev et al. [13] assumes the Gaussian distribution of results of physiological tests and requires the visual identification of a portion of the findings that are solely physiological.

The KOSMIC approach uses a combination of pathological and physiological test findings to estimate the distribution of physiological test results. This is achieved by a Box-Cox transformation and then Gaussian distribution fitting to a portion of the data that has been truncated. A Kolmogorov-Smirnov distance is computed between a truncated portion of the observed distribution and the Gaussian distribution. 'Various truncations of the observed distribution are employed, and the one with the smallest Kolmogorov-Smirnov distance is chosen as the data for healthy people'. The ‘estimated distribution’ may be utilised to determine the RI. KOSMIC is accessible as open-source software at https://gitlab.miracum.org/KOSMIC. A web-based tool accessible at https://KOSMIC.diz.uk-erlangen.de permits the usage of the KOSMIC application without the need for local installation [11].

The refineR algorithm employs a three-step inverse modelling approach. The first step is to identify the parameter search region and principal peak. In the second stage of the optimisation procedure, the approach employs a multi-level grid search for the optimal model parameters, such as λ (the power parameter), σ, μ and ‘P’ scaling factor, in order to estimate the optimal model for describing the underlying data. Finally, RIs may be obtained from the optimal model at the lowest cost. The refineR package (version 1.0.0), along with the getRI and resRI functions, was used to implement the refineR technique. To determine the 95% CIs, a bootstrap approach using 200 duplicates of the same database with random resampling was used [12,14]. Hoffman, KOSMIC and refineR’s reference ranges were compared to the published reference range in kit literature and standard textbooks.

## Results

A total of 63,469 results of TSH, 49,371 results of free T3 and 49,390 results of free T4 were retrieved from LIS in one and half years. We computed the RIs of TSH, as well as free T3 and T4, using Hoffman, KOSMIC and refineR algorithms. Table 1 summarises the RIs of free T3 and T4 and TSH in patients aged between 18 and 87 years along with a comparison IFU (instruction for use). It shows higher upper RIs for TSH using both KOSMIC and refineR algorithms, whereas the Hoffman method reveals comparable performance. Lower RIs of TSH, as well as both upper and lower RIs for free T4 and T3, revealed a good correlation with IFU (instructions for use) using Hoffman, KOSMIC and refineR methods.

**Table 1:** Comparison of RIs of thyroid hormones (serum TSH, free T3 and free T4) derived from KOSMIC and refineR with IFU TSH: thyroid-stimulating hormone; RI: reference interval; IFU: instructions for use.

Parameters	Reference range given in IFU	Reference range derived by computerised Hoffman’s method	Reference range derived by KOSMIC method	Reference range derived by refineR method
Serum TSH (mIU/L) (n=63496)	0.38-4.28	0.3-4.0	0.53-7.00; 90% CI - LL: 0.39-0.57; 90% CI - UL: 5.37-7.90	0.55-8.19; 95% CI - LL: 0.52-0.57; 95% CI - UL: 7.33-8.46
Free T3 (pg/mL) (n=49371)	2.1-4.4	2.4-5.0	2.37-5.22; 90% CI - LL: 2.35-2.57; 90% CI - UL: 7.33-8.46	2.11-5.15; 95% CI - LL: 2.04-2.63; 95% CI - UL: 5.17-5.32
Free T4 (ng/dL) (n=49350)	0.61-1.12	0.6-1.2	0.57-1.18; 90% CI - LL: 0.55-0.59; 90% CI - UL: 1.10-1.26	0.61-1.32; 95% CI - LL: 0.59-0.62; 95% CI - UL: 1.26-136

**Figure 1:**
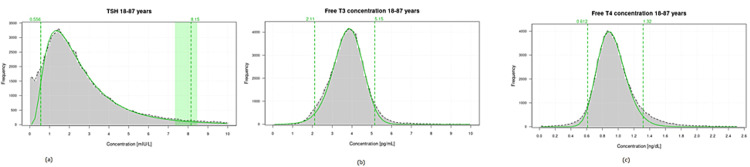
(a) Examples of estimated models of TSH hormones using refineR. (b) Examples of estimated models of free T3 hormones using refineR. (c) Examples of estimated models of free T4 hormones using refineR. TSH: thyroid-stimulating hormone.

**Figure 2:**
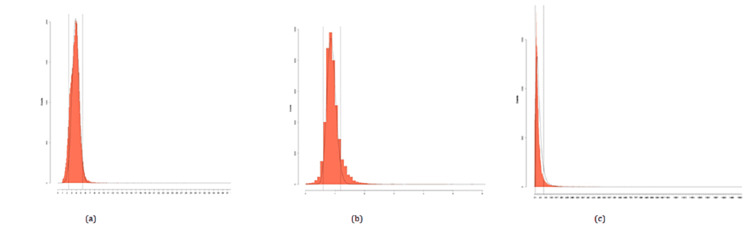
(a) Estimated distribution of physiological test results for TSH hormones. (b) Estimated distribution of physiological test results for free T3 hormones. (c) Estimated distribution of physiological test results for free T4 hormones TSH: thyroid-stimulating hormone.

**Figure 3:**
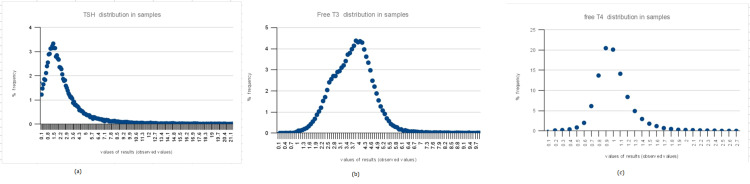
Estimation of (a)TSH, (b) free T3 and (c) free T4 by Hoffman’s method TSH: thyroid-stimulating hormone.

Figure 1 shows a graphical presentation of RIs using the refineR method. Figure 2 shows a graphical presentation of RIs using the KOSMIC algorithm, while Table 2 summarises the calculation of RIs for thyroid hormones using the KOSMIC algorithm along with the parameter μ, σ and upper and lower truncation limits, etc. Graphical presentation using the Hoffman method is shown in Figure 3.

**Table 2:** Calculation of RIs for thyroid hormones using KOSMIC algorithm TSH: thyroid-stimulating hormone; RI: reference interval.

	TSH concentration	Free T3 concentration	Free T4 concentration
N	63496	49371	49350
2.5% percentile	0.532845	2.37839	0.576794
50% percentile	2.04658	3.8005	0.849797
97.5% percentile	7.00022	5.22808	1.18847
λ	0.07	0.99	0.4
μ	0.734424	2.77787	-0.157573
σ	0.688931	0.717507	0.171608
T1	0.8	3.2	0.7
T2	3.7	4.1	0.9
Decimals	1	1	1
T1 min	0.05	0.05	0.05
T1 max	0.3	0.3	0.3
T2 min	0.7	0.7	0.7
T2 max	0.95	0.95	0.95
SD	0.8	0.8	0.8
Tolerance	1e-07	1e-07	1e-07

Histogram shows the distribution of the input data. The curve represents the estimated model which is the estimated distribution of the non-pathological test results. The vertical dashed lines show estimated lower and upper RIs.

## Discussion

ISO 15189:2012 recommends that laboratories regularly verify the in-use RIs when newly published RI analyses are present [15]. Because of the shortage of experience and knowledge to deal with data science, most of the laboratories in developing countries considerably hold back in estimating the potential of the available huge data. The methodology used by us relies on data science and retrieval of data from the laboratory information system of a tertiary care hospital’s laboratory, which receives samples from the entire region making a representation of all the ethnic groups.

RIs estimated using Hoffman, KOSMIC and refineR methods for free T3 and free T4 were compared to IFU RIs. For TSH, a difference was noted in RIs with both KOSMIC and refineR methods. Similar findings were also observed using refineR by Tatjana et al., who suggested that it may be caused by differences in the underlying population (e.g. age, ethnicity and time range, sex distribution and site of measurement) from the direct study and the indirectly analysed cohort [16]. The KOSMIC algorithm was used by Sibtain et al. to derive the RIs of TSH in the neonatal age period and found it very useful to derive RIs in paediatric populations in developing countries [17]. A similar study was conducted by Ma et al. using five indirect algorithms to verify RIs of thyroid hormones in older people and recommended the use of modified Hoffman, KOSMIC and refineR algorithms [18]. However, the performance of indirect methods is dependent on many characteristics of the input dataset, such as larger sample sizes (i.e. with around 5000 data points), and a pathological proportion of around 30% have shown better results [19].

In tertiary care hospitals, where a significant amount of abnormal patient data is present in the database, no statistical measure will match the standard method of measurement, i.e. direct method [20]. In the Hoffman method, linear points of a graph are taken by humans, whereas refineR and KOSMIC decisions are taken by computer after complex mathematics. So, the results of manual Hoffman and newer automated KOSMIC and refineR methods may not be agreed upon. All three indirect methods cannot be used for the establishment of RIs. They can only be utilised for verifying the RIs, because the study population used in indirect methods is the hospital population, whereas, in the direct method, we carefully select healthy patients to derive RIs. This study also emphasises that for the verification of RIs for TSH, refineR and KOSMIC are not good methods, especially data derived in tertiary care centres. The computerised Hoffman method described by katayev is a simple method and gives better results. The Hoffman method’s RI for TSH is more reliable even despite being susceptible to visual subjective bias [21]. This study also confirms that we cannot always apply statistical methods to biological data. If your data is skewed, such as TSH, results with the Hoffman method for verifying RIs are more reliable than the automated KOSMIC and refineR approach.

According to the PubMed database, this is the first study conducted on the Indian population to verify the RIs for TSH using a newer automated approach KOSMIC and refineR algorithms. However, the limitation of this study is that the Hoffman method is subjected to human bias, whereas KOSMIC and refineR’s performance declined for datasets with a high percentage (>50%) of pathological samples and a small number of datasets (n<5000). The main advantage of using indirect methods is to establish and/or verify RIs in the paediatric population in developing countries, which is planned in a future study using KOSMIC and refineR automated approaches. The performance of KOSMIC and refineR methods may be increased by taking a specific subset of hospital data (e.g. OPD patients who are not suffering from thyroid illness, such as skin OPD).

## Conclusions

Creating and utilising your thyroid hormones using RIs rather than a manufacturer interval provides a much better basis for diagnosing and considering treatment for thyroid dysfunction. Indirect laboratory approaches can be easily accepted in resource-constrained regions, and the RIs produced would provide a more precise comprehension of laboratory reports and facilitate patient care. The computerised Hoffman method proved better for parameters, such as TSH, in the hospital population compared to KOSMIC and refineR methods. If data distribution is extremely skewed, the results of the automated algorithms are not good.

## References

[1] Jones GR, Haeckel R, Loh TP (2018). Indirect methods for reference interval determination - review and recommendations. Clin Chem Lab Med.

[2] Mirjanic-Azaric B, Jerin A, Radic Z (2020). Thyroid stimulating hormone values of clinical decisions of hypothyroidism measurement by three different automated immunoassays. Scand J Clin Lab Invest.

[3] Haeckel R, Wosniok W, Streichert T (2021). Review of potentials and limitations of indirect approaches for estimating reference limits/ intervals of quantitative procedures in laboratory medicine. J Lab Med.

[4] Arzideh F, Özcürümez M, Albers E, Haeckel R, Streichert T (2021). Indirect estimation of reference intervals using ﬁrst or last results and results from patients without repeated measurements. J Lab Med.

[5] Hoffmann RG (1963). Statistics in the practice of medicine. JAMA.

[6] Bhattacharya CG (1967). A simple method of resolution of a distribution into gaussian components. Biometrics.

[7] Arzideh F, Wosniok W, Haeckel R (2011). Indirect reference intervals of plasma and serum thyrotropin (TSH) concentrations from intra-laboratory data bases from several German and Italian medical centres. Clin Chem Lab Med.

[8] Arzideh F, Wosniok W, Haeckel R (2010). Reference limits of plasma and serum creatinine concentrations from intra-laboratory data bases of several German and Italian medical centres: comparison between direct and indirect procedures. Clin Chim Acta.

[9] Arzideh F, Brandhorst G, Gurr E (2009). An improved indirect approach for determining reference limits from intra-laboratory data bases exemplified by concentrations of electrolytes. J Lab Med.

[10] Wosniok W, Haeckel R (2019). A new indirect estimation of reference intervals: truncated minimum chi-square (TMC) approach. Clin Chem Lab Med.

[11] Zierk J, Arzideh F, Kapsner LA, Prokosch HU, Metzler M, Rauh M (2020). Reference interval estimation from mixed distributions using truncation points and the Kolmogorov-Smirnov distance (kosmic). Sci Rep.

[12] Ammer T, Schützenmeister A, Prokosch HU, Rauh M, Rank CM, Zierk J (2021). refineR: a novel algorithm for reference interval estimation from real-world data. Sci Rep.

[13] Katayev A, Fleming JK, Luo D, Fisher AH, Sharp TM (2015). Reference intervals data mining: no longer a probability paper method. Am J Clin Pathol.

[14] (2022). The R Project for Statistical Computing. https://www.R-project.org.

[15] (2022). A International Organization for Standardization. ISO 15189: Medical laboratories—requirements for quality and competence. Geneva (Switzerland): International Organization for Standardization. https://www.iso.org/standard/56115.html#:~:text=ISO%2015189%3A2012%20can%20be,regulating%20authorities%20and%20accreditation%20bodies..

[16] Ammer T, Schützenmeister A, Rank CM, Doyle K (2023). Estimation of reference intervals from routine data using the refineR algorithm-a practical guide. J Appl Lab Med.

[17] Ahmed S, Zierk J, Khan AH, Jafri L, Majid H, Ghani F, Siddiqui I (2022). Reference intervals of serum TSH from mixed distributions using truncation points and the Kolmogorov-Smirnov distance. Clin Lab.

[18] Ma C, Zou Y, Hou L (2022). Validation and comparison of five data mining algorithms using big data from clinical laboratories to establish reference intervals of thyroid hormones for older adults. Clin Biochem.

[19] Ammer T, Schützenmeister A, Prokosch HU, Zierk J, Rank CM, Rauh M (2022). RIbench: a proposed benchmark for the standardized evaluation of indirect methods for reference interval estimation. Clin Chem.

[20] (2010). CLSI. Defining, Establishing, and Verifying Reference Intervals in the Clinical Laboratory; Approved Guideline - Third Edition. CLSI EP28-A3C. https://clsi.org/media/1421/ep28a3c_sample.pdf.

[21] Płaczkowska S, Terpińska M, Piwowar A (2022). Establishing laboratory-specific reference intervals for TSH and fT4 by use of the indirect Hoffman method. PLoS One.

